# Senescence-like Phenotype After Chronic Exposure to Isoproterenol in Primary Quiescent Immune Cells

**DOI:** 10.3390/biom14121528

**Published:** 2024-11-28

**Authors:** Michael Laumann, Philipp Palombo, Judy Fieres, Mara Thomas, Gabriele Saretzki, Alexander Bürkle, Maria Moreno-Villanueva

**Affiliations:** 1 Electron Microscopy Center, University of Konstanz, 78457 Konstanz, Germany; michael.laumann@uni-konstanz.de; 2 Molecular Toxicology Group, Department of Biology, University of Konstanz, 78457 Konstanz, Germany; philipp.palombo@uni-konstanz.de (P.P.); judy.salzwedel@web.de (J.F.); mara.thomas@roche.com (M.T.); alexander.buerkle@uni-konstanz.de (A.B.); 3 Biosciences Institute, Campus for Ageing and Vitality, Newcastle University, Newcastle upon Tyne NE4 5PL, UK; gabriele.saretzki@newcastle.ac.uk; 4 Human Performance Research Centre, Department of Sport Science, Box 30, University of Konstanz, 78457 Konstanz, Germany

**Keywords:** DNA strand breaks, catecholamine, isoproterenol, cellular senescence, β-galactosidase activity

## Abstract

Chronic stress is associated with a higher risk for carcinogenesis as well as age-related diseases and immune dysfunction. There is evidence showing that psychological stress can contribute to premature immunosenescence. Therefore, the question arose whether chronic exposure to catecholamine could drive immune cells into senescence. Peripheral blood mononuclear cells were isolated from whole blood. After repeated ex vivo treatment with isoproterenol, an epinephrine analog, well-established senescence biomarkers were assessed. We found (i) DNA double-strand break induction, (ii) telomere shortening, (iii) failure to proliferate, (iv) higher senescence-associated β-galactosidase activity, (v) decreases in caspases 3 and 7 activity, and (vi) strong upregulation of the proteoglycan versican accompanied by increased cellular adhesion suggesting the induction of a senescence-like phenotype. These results emphasize the complexity of the effect of isoproterenol on multiple cellular processes and provide insights into the molecular mechanisms of stress leading to immunosenescence.

## 1. Introduction

The human body’s reaction to psychological stress results in a rapid activation of the Sympathetic–Adreno–Medullar (SAM) axis and is accompanied by the release of the stress hormones epinephrine and norepinephrine from the adrenal glands [1,2,3]. During a stress response, the plasma concentration of epinephrine and norepinephrine increases up to 60 times, with a predominance of epinephrine (70%) over norepinephrine (30%) [4]. Epinephrine activates β-adrenergic (βAR) receptors, a family of G-protein-coupled receptors (GPCRs), causing a significant increase in lymphocyte counts, changing lymphocyte subpopulations [5,6,7,8], and decreasing lymphocytes’ mitogen sensitivity [5]. This stress response is adaptive to prepare the body to handle the challenges presented by stressors. However, prolonged exposure to chronic stress or experiencing frequent episodes of acute stress can lead to several health problems [9] including deficits in cellular immunity [10], cardiac hypertrophy, stroke, coronary artery disease, and heart failure [11].

Isoproterenol, also known as isoprenaline, is a sympathomimetic medication that targets βAR and therefore can be used to relieve bronchoconstriction [12] and treat bradyarrhythmias [13]. Isoproterenol also induces the vasodilatation of some peripheral blood vessels, especially those of skeletal muscles, causing a drop in blood pressure [12]. Epinephrine (also known as adrenaline) and isoproterenol are both potent β2AR agonists producing similar levels of cAMP in lymphocytes [14]. In scientific research, isoproterenol is used as epinephrine mimetic for investigating cellular mechanisms behind βAR activation under defined conditions [15,16,17,18,19]. In vivo, acute and chronic β2AR stimulation with isoproterenol seems to have opposing effects on circulating lymphocyte number and subset distribution in humans [20], but despite research efforts, the underlying molecular mechanisms of long-term or repeated βAR stimulation, especially in immune cells, are still poorly understood. Interestingly, several biomarkers of human aging have been associated with chronic psychological stress, such as the N-glycosylation profile [21], telomere shortening [22], or the accumulation of DNA damage [23]. It is known that due to damage accumulation, cells stop proliferating and can acquire the so-called “senescence-associated secretory phenotype” (SASP) [24]. Cellular senescence can be triggered by several factors including a gradual loss of telomeres [25] or DNA damage accumulation [26] leading to a persistent DNA damage response (DDR) [27], irreversible resistance to growth or proliferation factors [28], distinct metabolic activity and dramatic changes in cell morphology [29,30,31]. Therefore, the most established biomarkers of cellular senescence are those involved in telomere shortening, DDR, cell cycle regulation, structural changes, and enzymatic activities, such as β-galactosidase activity or apoptosis resistance [32,33].

In this work, we used isoproterenol to investigate whether experiencing frequent episodes of acute stress (the repeated stimulation of βAR ex vivo) would drive peripheral blood mononuclear cells into senescence.

## 2. Materials and Methods

### 2.1. Isolation of PBMC and T Cells

Venous blood was drawn from healthy donors using S-Monovettes containing an anticoagulant (Sarstedt, Nümbrecht, Germany). The selection of volunteers was performed in accordance with the Declaration of Helsinki, and ethical approval was obtained from the Ethics Committee of the University of Konstanz. Signed informed consent was obtained from each subject. Peripheral blood mononuclear cells (PBMCs) were isolated using Biocoll^®^ density centrifugation (Biochrom-Merck, Darmstadt, Germany). Isolated cells were resuspended in the medium for further analysis. The MACSxpress^®^ PAN T Cell Isolation Kit (Miltenyi Biotec, Bergisch Gladbach, Germany) was used for the isolation of T lymphocytes. Thereby, non-target cells were removed by specific immunolabeling using magnetic beads, while unlabeled lymphocytes were isolated. The isolation of T cells was performed according to the manufacturer’s instructions (Miltenyi Biotec, Bergisch Gladbach, Germany). The remaining erythrocytes were additionally eliminated via the MACSxpress^®^ Erythrocyte Depletion Kit (Miltenyi Biotec, Bergisch Gladbach, Germany), as well according to the protocol provided by the manufacturer. The cell suspension was centrifuged (4 °C, 300× *g*, 10 min), the supernatant was discarded, and the pellet was resuspended in the medium. Cell concentration and viability were determined via Casy Cell Counter TT (Innovatis, Berlin, Germany).

### 2.2. Isoproterenol Treatment

PBMCs were treated repeatedly with freshly prepared isoproterenol (Sigma Aldrich-Merck, Darmstadt, Germany). Isoproterenol was followed by 24 h incubation at 37 °C in a serum-free medium (TexMACS medium (Miltenyi Biotec, Bergisch Gladbach, Germany)) or an RPMI-1640 medium, both containing 100 U/mL penicillin and 100 mg/mL streptomycin (Invitrogen, Carlsbad, CA, USA). Cell concentration was adjusted to 2–4 × 10^6^ cells/mL. Isoproterenol was administrated either 1×, 4×, or 8× in time intervals of 1 h for the 4× treatment or 30 min for the 8× treatment. Treatment intervals of 30 and 60 min were defined in order to allow for the beta-adrenergic receptor to recover after isoproterenol binding and internalization. Receptor recovery of 85% after 20 min has been reported [34]. Each single treatment consisted of a final concentration of 10 μM isoproterenol. The cells were incubated at 37 °C in a shaking water bath (shaking frequency 50/min, stroke length 22 mm) for 24 and 48 h. Cell death was monitored using Casy Cell Counter TT (Innovatis, Berlin, Germany).

**Figure 1:**
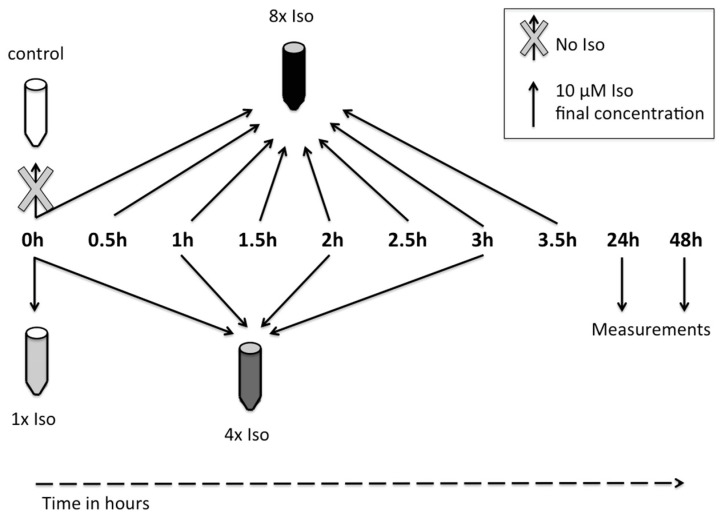
Schematic representation of chronic isoproterenol treatment. Isoproterenol was added to the cells 1× (0 h), 4× (0 h, 1 h, 2 h, and 3 h), or 8× (0 h, 0.5 h, 1 h, 1.5 h, 2 h, 2.5 h, 3 h, and 3.5 h). Each isoproterenol treatment led to a final concentration of 10 µM in medium. Measurements were performed after 24, 48, and 72 h incubation at 37 °C.

Chronic stimulation of the β2-AR was gained by repeated isoproterenol administration. Cells were treated 1×, 4×, and 8× with isoproterenol and incubated at 37 °C for 24, 48, and 72 h (Figure 1).

### 2.3. DNA Double-Strand Breaks

For the detection of DNA strand breaks, γH2AX immunostaining was performed. In a 2 mL reaction tube, 4 × 10^6^ cells/mL were resuspended in 400 μL of the cold RPMI-1640 medium. For fixation, 400 μL of ice-cold 100% analytical ethanol (−20 °C) was added to the cell suspension and inverted several times. Subsequently, cells were incubated at 4 °C for one hour. Then, 900 μL of cold FACS buffer was added and mixed thoroughly. After 15 min of incubation at 4 °C, cells were centrifuged at 4 °C and 750× *g* for 5 min. The pellet was resuspended with 660 μL of cold FACS buffer and transferred into a 96-well plate in triplicates with 200 μL of each cell suspension. Next, the cells were centrifuged at 4 °C and 750× *g* for 5 min. After removing the supernatant, a 100 μL antibody solution of the primary antibody (Rabbit, Cell Signaling Technology, Germany; 1:400) was added and the plate was stored at 4 °C for overnight incubation. Afterward, the cells were centrifuged at 4 °C with 750× *g* for 5 min, and the pellet was washed with 200 μL of the FACS buffer. Subsequently, 100 μL of the second antibody (1:1000 Alexa Fluor^®^ 488 Cell Signaling Technology, Danvers, MA, USA) was added, which was labeled with fluorochrome and incubated at 37 °C for 45 min in the dark. Finally, the cells were washed twice with 200 μL of the FACS buffer and transferred into FACS tubes. The samples were measured immediately via flow cytometry (BD FACSVerseTM Flow Cytometer, Becton Dickinson GmbH, Heidelberg, Germany)

### 2.4. Telomere Length Determination

The quantification of absolute telomere length in PBMC was performed according to a protocol from O’Callaghan with slight modifications [35]. Genomic DNA was isolated using the QIAGEN AllPrep DNA/RNA/Protein Mini Kit (QIAGEN, Duesseldorf, Germany). All steps were performed according to the manufacturer’s handbook with slight modifications: the incubation period of the buffers was extended up to two minutes to increase DNA yield and 2 × 50 µL Buffer EB was used for elution. The concentration and purity of the genomic DNA were determined by 260/280 UV spectrophotometry. A concentration of 5 ng/µL of DNA was stored at −20 °C until further usage. To quantify absolute telomere length, a standard curve of the control gene 36B4, which encodes the acidic ribosomal phosphoprotein P0, was generated in a serial dilution range from 100 to 10^−5^ (concentration of 200 pg to 2 × 10^−4^ pg oligomer). An additional standard curve of the synthesized oligonucleotide “Telomere standard” (TTAGGG repeated 14 times) was introduced by a serial dilution range from 100 to 10^−5^ (concentration of 62.5 pg to 6.25 × 10^−4^ pg oligomer). Moreover, 20 ng of Plasmid DNA (courtesy of Dr. Hanf) was added to each standard to maintain a constant 20 ng of total DNA per reaction. DNA of the 1301 lymphoblastic cell line (Sigma Aldrich-Merck, Darmstadt, Germany) was used as the positive control (pos) for long telomeres, and DNA from a HeLa cell line (courtesy of Dr. Karabatsiakis) was used as the negative control (neg) for short telomeres. The NTC sample (non-template control) was included in all reactions. All samples were run in technical triplicates. PCR reactions were run on the Bio-Rad CFX connect instrument (Bio-Rad, Feldkirchen, Germany). Cycling conditions (for both telomere and 36B4 amplicons) are as follows: 2 min at 95 °C, followed by 40 cycles of 95 °C for 15 s, 60 °C for 1 min, followed by a 1 min at 50 °C step, followed by a 1 min at 95 °C step, followed by a dissociation (or melt) curve, with a temperature gradient from 50 °C to 95 °C with an increase of 0.5 °C every 15 s. Quality control for all values was performed by excluding all samples with a difference in standard deviation greater than the 1 Cq- value. All samples were normalized to the positive control of one representative run. For the quantification of absolute telomere length, the SCG was divided by two since it has two copies in the human genome (diploid). Then, this value is used to normalize the values obtained for telomere length. The T/SCG ratio is further divided by 92 to obtain the mean absolute length of each human telomere.

### 2.5. Telomerase Activity Measurements Using TRAP ELISA

Total protein was extracted from frozen PBMC pellets using the CHAPS buffer, the protein was quantified using the Bradford reagent, and 500 ng of the protein was used for each sample. PCR was run at 30 cycles and the ELISA was measured at 450 nm on a plate reader (FLUOstar Omega, BMG Labtech, Offenberg, Germany).

The measurement of telomerase activity was performed using the Telo-TAGGG Telomerase PCR ELISA (Roche, Basel, Switzerland), according to the manufacturer’s handbook. The absorbance of wells without any lysate (background) was subtracted from the values of the lysates.

### 2.6. Proliferations Analysis

Cells were treated with isoproterenol in the TexMACS medium. The capacity of the cells to proliferate was determined after stimulation with Phytohemagglutinin-L (PHA-L, Sigma Aldrich-Merck, Darmstadt, Germany). Cell proliferation was assessed using the EdU Flow Cytometry Kit 488 Baseclick (Baseclick, Munich, Germany). To the 1 mL cell suspension (2 × 10^6^ cells/mL), 5 µL of PHA (1 mg/mL) was added and incubated at 37 °C. After 24 h of isoproterenol treatment, cells were incubated at 37 °C for another 24 and 48 h. Then, 1 µL of EdU was added to the cells 24 h before staining for flow Cytometry. Further steps were performed according to the manufacturer’s instructions except that 250 µL, instead of 500 µL, of “click assay cocktail” was used.

### 2.7. Gene Expression

Cells were treated with isoproterenol in the RPMI medium. The levels of mRNA were quantified by reverse transcription quantitative polymerase chain reaction (RT-qPCR) with custom-made Prime PCR Array plates (Bio-Rad, Feldkirchen, GER), and each well contained a lyophilized validated and specific primer pair for one gene. RNA was isolated using the DNA/RNA/Protein Mini Kit (Qiagen, Duesseldorf, Germany). Lysates were homogenized with the QIAshredder Homogenizer (Qiagen, Duesseldorf, Germany). After isolation, on-column DNase digestion was performed using the RNase-Free DNase Set (Qiagen, Duesseldorf, Germany). RNA was transcribed to cDNA with the iScript Advanced cDNA Synthesis Kit for RT-qPCR (Bio-Rad, Feldkirchen, Germany). Every reaction mix contained 190 ng of RNA, and all steps were performed according to the manufacturer’s instructions. cDNA was stored at −20 °C until qPCR was performed according to the instructions of the Prime PCR Assay (Bio-Rad, Feldkirchen, Germany). Quality controls for DNA contamination, RNA quality, reverse transcription, and PCR reaction, as well as two reference genes (GAPDH and HPRT1), were included. Thermal cycling and fluorescence detection were performed with the CFX Connect Real-Time PCR Detection System (Bio-Rad, Feldkirchen, Germany). The cycling protocol was as follows: activation temperature of 95 °C for 2 min for one cycle; denaturation temperature for 5 s and annealing/extension ay 60 °C for 30 s for 40 cycles; Melt Curve SYBR Green temperature of 65 °C to 95 °C (0.5 °C increments) 5 s/step for one cycle. The relative transcription of a gene in the treated sample compared to the control sample was calculated according to the ΔΔCq-method.

### 2.8. Senescence-Associated β-Galactosidase Activity

Cells were treated with isoproterenol in the TexMACS medium. Senescence-associated β-galactosidase activity was determined using the Beta-Glo^®^ Assay System (Promega, Walldorf, GER). A calibration curve was performed using commercially obtained recombinant β-galactosidase from *E. coli* (~140 U/mg, Sigma Aldrich-Merck, Darmstadt, Germany). Then, 25 μL containing 1 × 10^4^ cells per measuring point was used for staining. Positive control cells were treated with different concentrations of H_2_O_2_. The β-galactosidase assay was performed according to the manufacturer’s instructions.

### 2.9. Cell Adhesion

After 24 h, isoproterenol treatment cells were adjusted to 5 × 10^6^ cells/mL. Thereafter, 10 µL of the cell suspension was placed on Nunc Thermanox coverslips (12 mm) (Sigma Aldrich-Merck, Darmstadt, Germany) placed in 12-well plates. The cells were incubated at 37 °C for 20 min for sedimentation and then fixed with 4% formaldehyde and 0.4% glutardialdehyde in 0.1 M Sodium-Cacodylate buffer with 0.09 M sucrose, 0.1 M MgCl_2_, and 0.1 M CaCl_2_. Cells were washed with the buffer, and a secondary fixation with 1% osmium tetroxide (OsO_4_) in the buffer for 60 min at 4 °C in the dark was carried out. Afterward, cells were washed in the buffer again and dehydrated in increasing concentrations of ethanol (30% up to absolute ethanol) for 10 min each step. Samples were critical-point-dried in CO_2_ in a Balzers CPD030 (Balzers, Lichtenstein, Bergisch Gladbach, Germany) and sputter-coated with a 6 nm thick layer of gold/palladium in Balzers SCD030 (Balzers, Lichtenstein). Micrographs were produced using Zeiss Auriga FESEM (Zeiss, Oberkochen, Germany).

### 2.10. Activity of Caspases 3 and 7

The apoptosis of previously stressed PBMCs was investigated by detecting caspase 3 and 7 activities according to the luminescent Caspase-Glo^®^ 3/7 Assay (# G8090/G8091, Promega, Walldorf, Germany). Thereby, a luminogenic caspase 3 and caspase 7 substrate is cleaved to aminoluciferin only in the presence of cellular caspase 3 and 7 activity, resulting in the generation of light following the luciferase reaction. Luminescence is proportional to the amount of caspase activity present. Caspases 3 and 7 activity was detected in 1 × 10^4^ cells/well and assessed as indicated by the manufacturer. Treatment with 100 μM etoposide for 1 h was used as a positive control. Etoposide was added to the cell suspension 1 h before cells were processed for the detection of caspase activity.

### 2.11. Statistics

Two-way-repeated measured ANOVA followed by Dunnett’s multiple comparisons test was performed to address the statistical significance of both treatment and time, as well as the treatment × time interaction, in the percentage of γH2AX-positive PBMCs. A non-parametric Friedman test was performed for the dose response (line above all bars) and Dunn’s multiple comparison test was performed for each treatment compared to the untreated control (asterisks above corresponding bars) for all other measurements except for gene expression. For gene expression analysis, a non-parametric Wilcoxon signed rank test compared the relative gene expression of treated cells to a control value = 1. The error bars indicate the standard error of the mean (SEM) * *p* < 0.05, ** *p* < 0.01, *** *p* < 0.005. Statistical analyses were performed using Prism 8 (GraphPad Software, MA, USA)

## 3. Results

We previously showed that repeated treatment with isoproterenol induced DNA single-strand breaks and reduced PARP1 protein and its activity [36,37]. In this study, we addressed the hallmarks of senescence in PBMCs in response to repeated exposure to isoproterenol.

### 3.1. Isoproterenol Induces Telomere Shortening but Does Not Change Telomerase Activity

**Figure 2:**
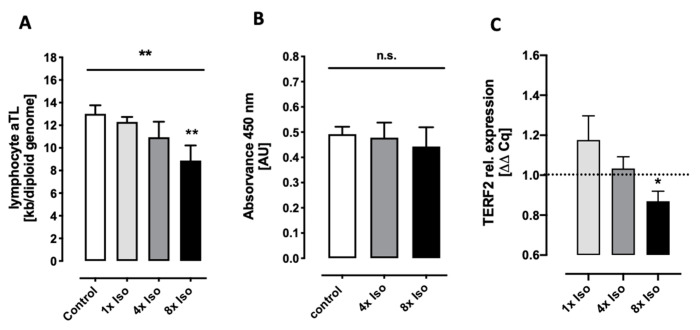
Measurement of telomere length, telomerase activity, and expression of TERF2 gene in isoproterenol-treated PBMCs. All measurements were performed 24 after the first treatment. (**A**). Absolute telomere length of lymphocytes treated with either 0×, 1×, 4×, or 8× isoproterenol. With increases in isoproterenol treatment, the kb per diploid genome decreases with statistical significance. Data for each treatment consist of *n* = 8 independent experiments. (**B**) Activity of telomerase in total protein extracts of PBMCs treated with either 0×, 4×, or 8× isoproterenol. Telomerase activity is not significantly different between the treatments and the control. Data for each treatment consist of *n* = 10 independent experiments. (**C**) Expression of the telomeric repeat binding factor 2 (TERF2) gene after single (1×) and repeated (4× and 8×) isoproterenol treatment. Relative gene expression significantly decreases after 8x isoproterenol treatment. Data for each treatment consist of 1× *n* = 3, 4× *n* = 3, and 8× *n* = 11 independent experiments. Statistical test for A and B: non-parametric Friedman test for dose response (line above all bars) and Dunn’s multiple comparison test for each treatment compared to untreated control (asterisks above corresponding bars). C: Statistical test for C: Wilcoxon signed rank test compared to control value = 1. A, B, and C error bars indicate standard error of the mean (SEM) * *p* < 0.05, ** *p* < 0.01.

Telomere shortening and damage are known causes of cellular senescence and aging [38]. Since GGG sequences are more susceptible to oxidative stress, telomere shortening without replication can happen just by the induction of double-strand breaks through oxidative stress [39], but it has been shown that the rate of telomere shortening is accelerated when cells are exposed to genotoxic stresses (e.g., reactive oxygen species (ROS)) [40]. Studies also showed that telomeric lesions remain unrepaired for several months both in vitro and in vivo [41,42], and up to half of the DNA damage in stress-induced senescence is located at telomeres irrespective of telomerase activity [42]. Furthermore, a link between telomere shortening and transcriptional changes occurring in senescent cells has been reported showing that the shelterin protein TERF2 is downregulated in senescent cells [43] and, in contrast, its overexpression delays senescence [44]. Moreover, the inhibition of TERF2 promotes T-cell telomere attrition and telomeric DNA damage that accelerates T-cell senescence [45]. Accordingly, we found telomere shortening and the downregulation of TERF2 after isoproterenol treatment. Telomerase activity is generally repressed in quiescent cells [46,47]. Therefore, as expected, we did not detect any significant changes in telomerase activity (Figure 2).

### 3.2. Isoproterenol Induces DNA Double-Strand Breaks and Reduced Caspase 3 and 7 Activity

**Figure 3:**
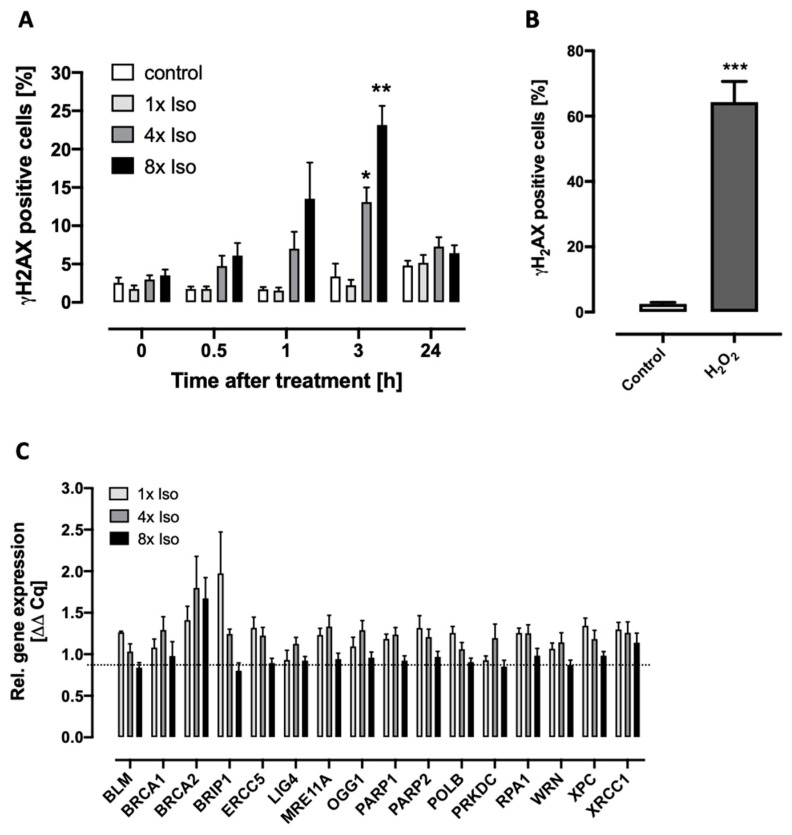
Analysis of DNA damage and expression of DNA repair genes. (**A**) DNA strand breaks detected via **γ**H2AX immunofluorescence flow cytometry: Percentage of **γ**H2AX-positive PBMCs treated with either 0×, 1×, 4×, or 8× isoproterenol was quantified after 0.5, 1, 3, and 24 after isoproterenol treatment. Data for each treatment consist of *n* = 5 independent experiments. The percentage of positive cells significantly changes with time and treatment. Two-way repeated-measure ANOVA shows statistical significance of both treatment (*p* = 0.0035) and time (*p* = 0.0065), as well as treatment × time interaction (*p* = 0.0069). Asterisks indicate statistical significance after Dunnett’s multiple comparisons test. (**B**) Internal control for **γ**H2AX immunofluorescence. PBMCs were treated with 1mM H_2_O_2_ for 10 min, stained, and counted by flow cytometry with treated cells. Paired Student’s *t*-test shows significant increase in the percentage of **γ**H2AX-positive cells after treatment. Data for each treatment consist of *n* = 13 independent experiments. (**C**) Expression of genes involved in DNA strand breaks repair 24 h after 0×, 4×, or 8× isoproterenol treatment relative to non-treated cells. Data consist of 1× *n* = 3, 4× *n* = 8, and 8× *n* = 16 independent experiments. Isoproterenol treatment did not significantly affect gene expression. A, B, and C error bars indicate standard error of the mean (SEM) * *p* < 0.05, ** *p* < 0.01, *** *p* < 0.005.

Usually, DNA single-strand breaks can be rapidly repaired by cellular repair mechanisms. However, unrepaired DNA single-strand breaks (SSB) can be converted into highly toxic DNA double-strand breaks (DSB) [48,49] leading to cell death, cancer, or senescence. DNA repair deficiency is known to accelerate aging in vivo [50], and the transcriptional repression of DNA repair genes is widely recognized as a cause of cellular senesce [51]. Isoproterenol treatment increases the percentage of cells with DNA double-strand breaks up to 25% in a time- and dose-dependent manner. However, only 7% of cells showed residual DNA strand breaks after 24 h, indicating successful DNA repair. Moreover, the expression of key genes involved in DNA single- and double-strand break repair did not significantly change (Figure 3).

**Figure 4:**
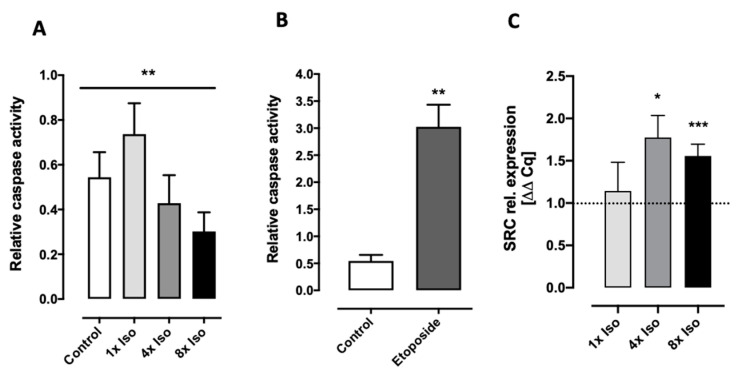
Determination of caspase activity and SRC gene expression. (**A**) Caspase activity and gene expression measurements were performed 24 h after the first treatment. Data for each treatment consist of *n* = 6 independent experiments. Non-parametric Friedman test shows significant changes in caspase activity for dose response (line above all bars), while Dunn’s multiple comparison test for each treatment did not reach statistical significance when compared to untreated cells. Asterisks indicate statistical significance after Dunnett’s multiple comparisons test. (**B**) Internal control for caspase activity assay. PBMCs were treated with 1mM etoposide for 10 min and analyzed parallel to treated cells. Paired Student’s *t*-test shows significant increase in caspase relative activity after etoposide treatment. Data represent means with a standard error of the mean of *n* = 6 independent experiments. (**C**) Expression of the SRC kinase gene after single (1×) and repeated (4× and 8×) isoproterenol treatment. Relative gene expression significantly increases after 4x and 8x isoproterenol treatment (Wilcoxon signed rank test compared to control value = 1, asterisks above corresponding bars). Data for each treatment consist of 1× *n* = 3, 4× *n* = 8, and 8× *n* = 16 independent experiments. A, B, and C error bars indicate standard error of the mean (SEM) * *p* < 0.05, ** *p* < 0.01, *** *p* < 0.005.

Senescent cells are largely resistant to apoptosis and display an upregulation of anti-apoptotic pathways [52,53]. The inhibition of caspases blocks intrinsic apoptosis at many steps of the pathway, leaving senescence as an alternative cell fate [54]. Here, we show that repeated treatment with isoproterenol decreases caspase 3 and 7 activity and increases tyrosine-protein kinase SRC gene expression in a dose-dependent manner (Figure 4).

### 3.3. Isoproterenol Inhibits PHA-Induced Cell Proliferation

**Figure 5:**
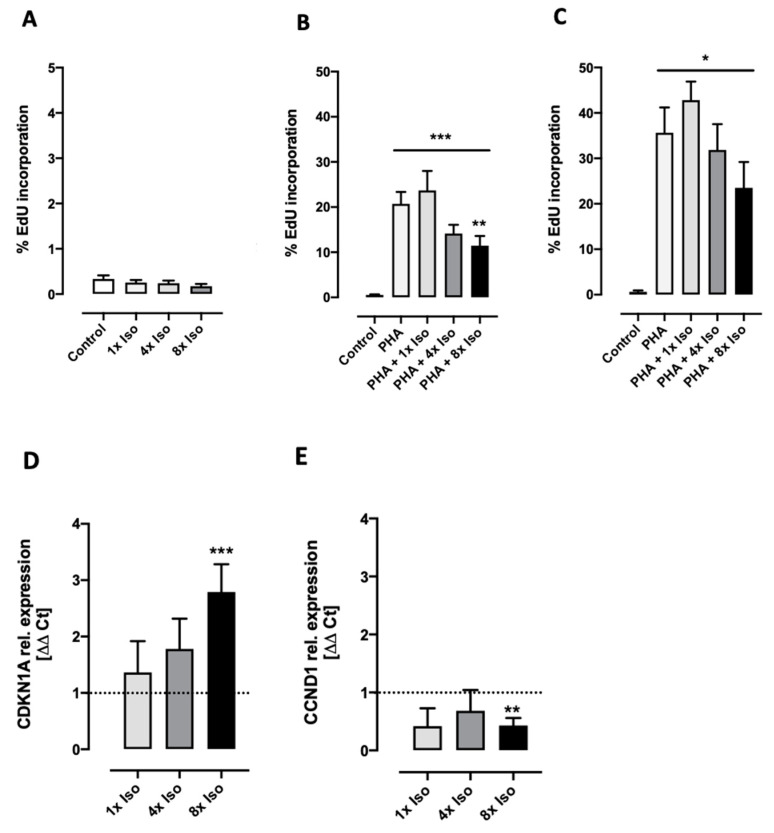
Isoproterenol-dependent inhibition of proliferation in PHA-stimulated PBMCs. (**A**) DNA synthesis was not induced by isoproterenol treatment. After 48 (**A**,**B**) or 72 h (**C**) incubation, DNA synthesis was increased in PHA-stimulated cells but reduced if cells were previously treated with isoproterenol. Data for each treatment consist of *n* = 11 and *n* = 4 independent experiments for B and C, respectively. Non-parametric Friedman test shows significant changes in newly incorporated EdU for dose response (line above all bars), while Dunn’s multiple comparison test was performed to compare each treatment to untreated cells (asterisks above corresponding bars). Expression of CDKN1A (**D**) and CCND1 (**E**) after 24 h after single (1×) and repeated (4× and 8×) isoproterenol treatment. CDKN1A relative gene expression significantly increases while CCDN1 significantly decreases after 8x isoproterenol treatment (Wilcoxon signed rank test compared to control value = 1, asterisks above corresponding bars). Data for each treatment consists of *n* = 3 for 1× treated and 4× *n* = 8 and 8× *n* = 16 independent experiments. A, B, and C error bars indicate standard error of the mean (SEM) * *p* < 0.05, ** *p* < 0.01, *** *p* < 0.005.

Senescence is characterized by an irreversible cell-cycle arrest mainly in the G1 phase, which prevents the proliferation of damaged cells [55,56]. By contrast, cellular quiescence is a reversible growth arrest state where cells are not actively dividing but retain the capacity to re-enter the cell cycle upon receiving an appropriate stimulus [57,58]. T cells are actively maintained in a quiescence state. However, antigen binding drives T cells to exit quiescence, promoting cell proliferation and differentiation [59]. In order to investigate whether isoproterenol inhibits proliferation, the mitogen lectin phytohemagglutinin (PHA) was used as a stimulus to induce cell-cycle activity [60]. Significant increases in the percentage of dividing cells of 19.3% (A) and 42.8% (B) were observed after 48 and 72 h after incubation with PHA, respectively. Pretreatment with isoproterenol significantly decreased the percentage of proliferating cells in a dose-dependent manner. As expected, this was accompanied by the dysregulation of cell-cycle regulatory genes. While CDKN1A (p21) was upregulated, CCND1 (cyclin D1) was downregulated (Figure 5). Reciprocal regulation of these genes clearly indicates cell-cycle arrest [61,62,63].

### 3.4. Isoproterenol Induces Cell Adhesion

**Figure 6:**
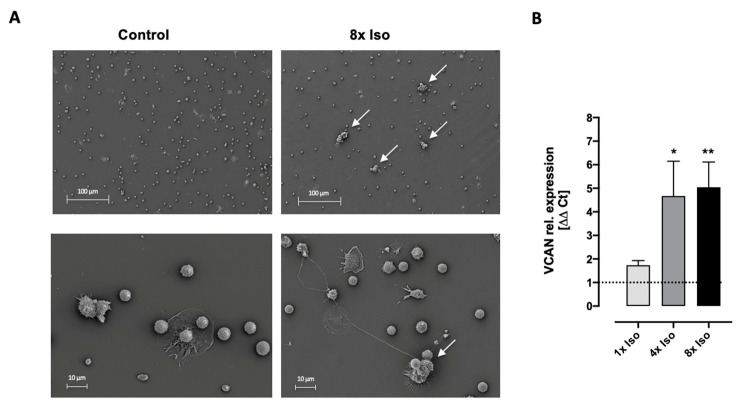
Treatment of isoproterenol in PBMCs induces cellular adhesion. (**A**) Representative scanning electron microscope pictures from cultured PBMCs 24 h after 8× isoproterenol treatment. Arrows show cell adhesion. (**B**) Normalized gene expression of VCAN in PBMCs after single (1×) and repeated treatment (4× and 8×) with isoproterenol. VCAN relative gene expression significantly increased 24 h after 4× and 8× isoproterenol treatment (Wilcoxon signed rank test compared to control value = 1, asterisks above corresponding bars). Data for each treatment consist of *n* = 3 for 1× treated and 4× *n* = 8 and 8× *n* = 16 independent experiments. Error bars indicate standard error of the mean (SEM) * *p* < 0.05, ** *p* < 0.01.

Senescence has long been closely associated with a hyper-adhesive cell phenotype [64,65]. Versican is an extracellular matrix (ECM) proteoglycan produced by stromal cells as well as leukocytes and is markedly increased in inflammation [66,67]. Here, we show an increase in cell adhesion after isoproterenol treatment (Figure 6A), which was accompanied by an increase in VCAN gene expression (Figure 6B).

### 3.5. Isoproterenol Induces Senescence-Associated β-Galactosidase Activity

**Figure 7:**
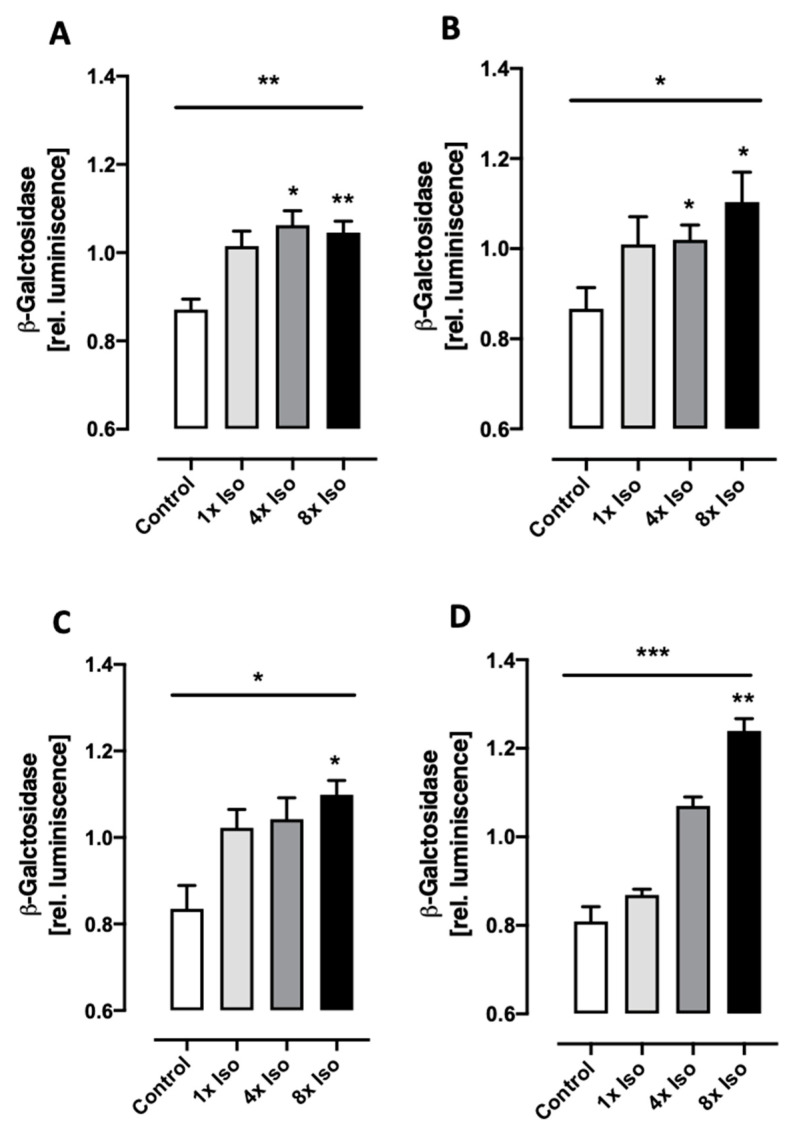
Senescence-induced beta-galactosidase activity. (**A**,**B**) PBMCs and (**C**,**D**) T cells. Luminescence (=amount of β-galactosidase) was measured after 24 h (**A**,**C**) and 48 h (**B**,**D**). Non-parametric Friedman test shows significant increase in luminescence in a dose-dependent manner (line above all bars), while Dunn’s multiple comparison test was performed to compare each treatment to the control untreated cells (asterisks above corresponding bars). Data for each treatment consist of *n* = 5 independent experiments. Error bars indicate standard error of the mean (SEM) * *p* < 0.05, ** *p* < 0.01, *** *p* < 0.005.

Senescent cells express β-galactosidase activity detectable at pH 6.0, so-called “senescence-associated β-galactosidase” activity (SA-βgal) [68,69,70]. SA-βgal is a manifestation of residual lysosomal activity at a suboptimal pH, which becomes detectable due to the increased lysosomal content in senescent cells [71]. Although SA-βgal might not be exclusive to senescence, this enzyme seems to be the most reliable biomarker for distinguishing senescent cells from quiescent ones [72]. Since lysosomal expansion is also a feature of macrophages [73,74], SA-βgal activity detected in PBMCs could be attributed to the proportion of macrophages present in PBMCs. Therefore, we measured SA-βgal activity in PBMCs (Figure 7A,B) as well as in isolated T cells (Figure 7C,D). Significantly increased SA-βgal activity was detected in both cell populations 24 and 48 h after isoproterenol treatment in a dose-dependent manner.

## 4. Discussion

The relative efficacy and potency of isoproterenol for generating cAMP in human lymphocytes is comparable to epinephrine. For this reason, isoproterenol is used as an epinephrine analog in in vivo and in vitro studies. At the cellular level, the acute activation of βAR results in the generation of cAMP as a second messenger. Thereafter, the activated βAR is phosphorylated, resulting in the binding of β-arrestin and leading to receptor desensitization [75]. However, prolonged stimulation of the βAR receptor through isoproterenol triggers DNA damage in vivo and in vitro [76]. DNA damage and the subsequent activation of the DNA damage response (DDR) pathways might contribute to the establishment and maintenance of cellular senescence [26]. Interestingly, emerging data suggest the involvement of G-protein-coupled receptors (GPCRs) and their associated proteins in the development of cellular senescence [77]. For example, cardiac senescence is involved in the process of pathological cardiac hypertrophy induced by isoproterenol [78]. Therefore, we asked the question of whether chronic exposure to isoproterenol would drive PBMCs into senescence. To answer this question, we relied on well-established senescence biomarkers.

There is evidence suggesting that telomeres act as molecular sensors of intrinsic and extrinsic stresses [79] and telomere damage can trigger cellular senescence [80,81]. If telomeric double-strand breaks cannot be repaired, DDR proteins remain permanently attached to the damage site and the cell enters a state of senescence [82]. We found that telomere shortening was induced by isoproterenol while telomerase activity was not affected (Figure 2A,B). These results are intriguing because telomeres can only shorten during cell division. Without cell division, telomeres only accumulate DNA damage, which is not translated into telomere shortening [83]. However, there might be other mechanisms of telomere shortening that appear as abrupt telomere shortening and might not need cell division [84]. In our study, since cells were not mitogenically stimulated and isoproterenol inhibits cell proliferation, we speculate that telomere shortening might occur due to other mechanisms [84]. Furthermore, it has been shown that the rate of telomere shortening is accelerated when cells are exposed to genotoxic agents (e.g., reactive oxygen species (ROS)) [40]. The accumulation of DNA damage in telomeres could be attributed to TERF2 deficiency since DNA repair within telomeres is suppressed by TERF2, and this mechanism is indispensable to the cell as it prevents telomere fusion due to NHEJ [82]. Indeed, we found slight TERF2 downregulation after isoproterenol treatment in a dose-dependent manner (Figure 2C).

As mentioned above, senescence can be also induced by DNA damage. Although our data show the induction of DNA double-strand breaks upon isoproterenol treatment, these are almost completely repaired after 24 h (Figure 3A). Furthermore, the expression of key genes involved in DNA repair did not change (Figure 3C). Therefore, either the time point 24 h after treatment was too late (or too early) to see expression changes, or the DNA repair mechanisms can deal with the relatively low amount of DNA damage triggered by isoproterenol. Interestingly, the induction of either apoptosis or senescence might be dependent on the level of damage. For example, sublethal stress and damage from internal and external sources such as H_2_O_2_, hyperoxia, tert-butylhydroperoxide, or UVB exposure can trigger human diploid fibroblasts (HDFs) and melanocytes to enter senescence, defined as “stress-induced premature senescence” (SIPS) [85]. With low DNA damage, the SRC-mediated activation of p38 critically promoted the expression of cell survival and senescence proteins, while high DNA damage failed to activate SRC, leading to the elevation of p53, the inhibition of p38, and apoptosis [86]. The inhibition of caspases blocks intrinsic apoptosis at many steps of the pathway, leaving senescence as an alternative cell fate [54]. In mice exposed to the DNA-damaging agent etoposide, pharmacologic inhibition of the tyrosine kinase SRC prevented the accumulation of senescent cells in tissues [86]. Furthermore, the interplay between Src and caspase 8 has been proposed as an apoptosis/survival functional switch [87]. Our data on gene expression show a significant upregulation of SRC (Figure 4C) and low DNA damage that remained unrepaired after 24 h (Figure 3A). In our previous study, isoproterenol-induced apoptosis seemed to be strongly subject-dependent, indicating that anti-apoptotic mechanisms may also play a role [37]. Here, we measured a clear decrease in caspases 3 and 7 activity after 4× and 8× isoproterenol treatment (Figure 4A). However, the role of adrenergic receptors in apoptosis is complex. While several authors showed an induction of apoptosis after receptor stimulation [88,89,90], others have reported an inhibition [91,92]. These differences might be due to cell type, short vs. prolonged stress exposure [93], and/or inter-individual variability [94,95,96].

The transition between cell-cycle phases of eukaryotic cells is controlled by a set of cyclin/cyclin-dependent kinase (CDK) complexes. The inhibitory effects on the activity of CDK-cyclin complexes may lead to cell-cycle arrest (temporary or permanent), differentiation, senescence, quiescence, or apoptosis [58,97]. Cells can undergo senescence in response to the overexpression of cell-cycle inhibitors such as p21 and p16 [98]. The cyclin-dependent kinase inhibitor p21 (CDKN1A) plays essential roles in the DNA damage response and is activated by DNA damage, inducing cell-cycle arrest, inhibiting DNA replication, and regulating apoptosis and transcription [99]. Stein et al. proposed that in late senescent cells, the inactivation of cyclin-dependent kinases and cell-cycle arrest is maintained through the combined effect of p16 and p21 [100]. Additionally, p21 might also be involved in the β2 adrenoreceptor-mediated inhibition of oligodendrocyte proliferation [101] and inhibits cyclin D1, promoting the onset of senescence induced by DNA damage [61]. Interestingly, cyclin D1 has also been associated with senescence in epithelial cells and fibroblasts [102,103]. Regarding the cell cycle, our results strongly indicate that previous treatment with isoproterenol inhibits PHA-induced cell proliferation (Figure 5B,C). G1 arrest is mediated by p21Waf1/Cip1 (p21), which inhibits G1 cyclin (CycD1 and CycE1)-bound CDKs [61]. Indeed, we found a decrease in the expression of cyclin D1 (CCND1) (Figure 5E) and an increase in the expression of cyclin-dependent kinase inhibitor 1A (CDKN1A) (Figure 5D).

The gene expression and or protein levels of p16 are frequently used as a senescence marker in human peripheral blood cells [104]. Although we observed a slight increase in the p16 protein after isoproterenol treatment, it was neither consistent nor significant. Previous studies have shown that the highest and most stable expression of p16 is observed in CD3+ T cells from older donors, which likely suggests the accumulation of senescent cells in peripheral blood over time [105]. Interestingly, isoproterenol-induced senescence in cardiomyocytes showed an increase in SA-β-GAL within 2 days of the isoproterenol treatment, but higher levels of proteins p16 and p21 were observed 7 days after the first isoproterenol infusion [78]. In this work, blood was drawn from young subjects and p16 was measured in non-stimulated cells. This could explain the lack of p16 upregulation in our experiments.

There is evidence of adrenergic modulation of lymphocyte migration and adhesion [94,106]. For example, isoproterenol induces integrin-mediated cell adhesion [107]. Furthermore, the dysregulation of adhesion molecules has also been associated with senescence and aging. The replicative senescence of vascular endothelial cells induced the increased expression of adhesion molecules, promoting monocytic adhesion [108]. Also, morphological cellular changes such as a flat large shape are the main features of the senescent phenotype [64]. Previous studies reported that beta-adrenergic stimulation also leads to lymphocyte aggregation [109]. Furthermore, experiments conducted in mouse cardiac fibroblasts showed increased versican expression after β1-adrenergic receptor stimulation [110]. Versican is one component of the extracellular matrix that can influence the ability of cells to proliferate, migrate, and adhere [111,112]. It is produced by leukocytes and modulates the innate immune response through interactions with other extracellular matrix molecules [66,67]. We found significant and strong upregulation of VCAN (Figure 6B) and increased cellular adhesion after isoproterenol treatment (Figure 6A). Interestingly, versican has been shown to inhibit apoptosis [67] and induce NF-κB pathway activation, DNA double-strand breaks, and telomere shortening [113], suggesting its involvement in the induction of cellular senescence. Indeed, VCAN was identified as a novel direct miR-126a-5p target that induces telomere shortening in bone mesenchymal stem cells (BMSCs), inducing senescence [113,114] and the silencing of VCAN, resulting in decreased expression of SA-βgal activity [113].

In contrast to proliferation-associated genes, the senescence marker SA-β-gal activity is expressed in senescent but not pre-senescent or quiescent fibroblasts, nor in terminally differentiated keratinocytes [69], although SA-βgal can be detected in a senescence-independent manner and the fraction of SA-βgal positive cells increases in aged tissues, consistent with the accumulation of senescent cells with age in vivo [69]. Therefore, SA-βgal remains tightly associated with the senescent phenotype and is a well-accepted biomarker of senescence [104,115]. Furthermore, SA-β-gal activity increases in isoproterenol-treated cardiomyocytes [78]. Therefore, we also investigated the effect of isoproterenol on SA-βgal activity. Macrophages and senescent cells are both characterized by an expanded lysosomal compartment and, therefore, SA-βgal is also a feature of macrophages [74,116]. Therefore, SA-βgal activity was measured in PBMCs as well as in isolated T cells. We found a small but significant increase in SA-βgal activity in PBMC treated 4x and 8x with isoproterenol (Figure 7A,B), and this difference was more pronounced in isolated T cells (Figure 7C,D).

## 5. Conclusions

**Figure 8:**
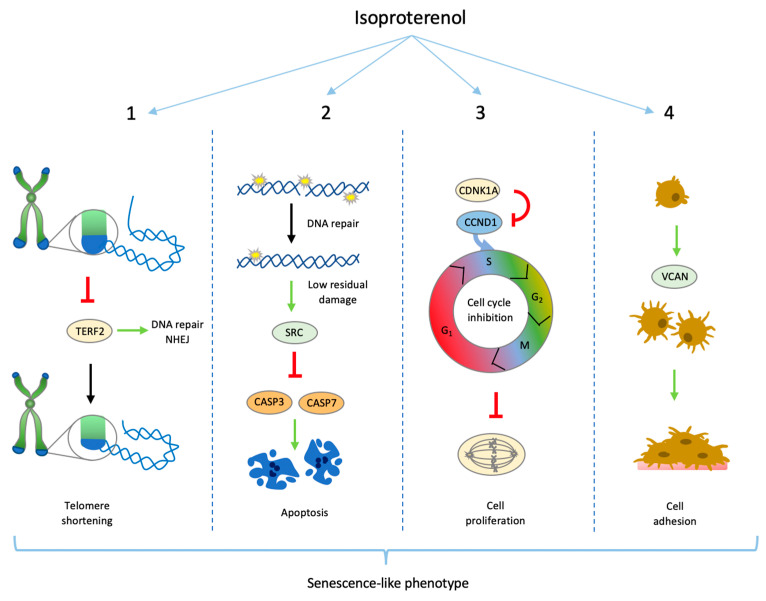
Schematic representation of main outcomes in the context of previously published results. (**1**). Inhibition of TNFR2, which might expose telomere to be recognized by non-homologous end-joining (NHEJ) repair proteins resulting in telomere shortening. (**2**). Low DNA damage induced SRC-mediated activation of p38, reducing CASP activity and promoting cell survival and senescence. (**3**). Upregulation of CDNK1A induces downregulation of CCND1, leading to suppression of cell proliferation after PHA stimulus. (**4**). Upregulation of VCAN induces cell adhesion.

Similar to isoproterenol-induced senescence in the development of cardiac hypertrophy [78], our data suggest the induction of a senescence-like phenotype in primary human blood peripheral cells treated with isoproterenol. The induction of telomere shortening, suppression of the cell cycle, inhibition of apoptosis, and increased cell adhesion are multiple consequences of repeated isoproterenol treatment. These findings, together with cell morphology changes and increased SA-βgal activity, enhance our understanding of how repeated acute stress can lead to immunosenescence (Figure 8). However, whether or not these effects are ß2 adrenergic receptor-mediated needs to be further investigated.

## 6. Limitations

Cellular senescence is reported to take, in general, several days to be completed [117,118]. Although cell-cycle arrest in cultured cells occurs within 24 h of damage, cells develop a full SASP > 5 days after senescence induction [119]. Therefore, a time-dependent assessment of some of the biomarkers, especially gene expression analyses, needs to be performed. Culture conditions might also have an impact on the results. For instance, serum albumin is essential for the in vitro growth of activated human lymphocytes [108,120], and we recently published that the ex vivo culturing of non-stimulated primary immune cells for longer than 24 h strongly increases isoproterenol-dependent cell death in RPMI-1640 without FCS [36]. Therefore, the TexMACS medium containing human albumin was used for experiments requiring longer incubation and for cell-cycle analyses. The effect of albumin on senescence biomarkers in lymphocytes needs to be further investigated. Last but not least, the activation of the adrenergic system has a profound effect on energy metabolism [121,122,123]. We previously showed that repeated treatment with isoproterenol reduces cAMP (as expected), NAD^+^, and ATP ex vivo [36,37]. Lowering the NAD^+^/NADH ratio results in ATP depletion, AMPK activation, and cell-cycle arrest [124], eventually involving mitochondrial respiration, which was not measured in this study.

## Data Availability

Data will be available upon request to Maria Moreno-Villanueva (maria.moreno-villanueva@uni-konstanz.de).
